# Urate‐lowering therapy in patients with hyperuricemia and heart failure: A retrospective cohort study using the UK Clinical Practice Research Datalink

**DOI:** 10.1002/clc.24297

**Published:** 2024-06-14

**Authors:** Steven J. Kiddle, Karolina Andersson Sundell, Shira Perl, Stephen Nolan, Magnus Bjursell

**Affiliations:** ^1^ Data Science & Advanced Analytics Data Science & Artificial Intelligence, R&D, AstraZeneca Cambridge UK; ^2^ Cardiovascular, Renal and Metabolic (CVRM) Evidence, BioPharmaceuticals Medical, AstraZeneca Gothenburg Sweden; ^3^ Late‐stage Development, Clinical, Cardiovascular, Renal and Metabolic (CVRM), BioPharmaceuticals R&D, AstraZeneca Gaithersburg Maryland USA; ^4^ Late‐stage Development, Clinical, Cardiovascular, Renal and Metabolic (CVRM), BioPharmaceuticals R&D, AstraZeneca Cambridge UK; ^5^ Global Medical Affairs, Clinical, Cardiovascular, Renal and Metabolic (CVRM), BioPharmaceuticals Medical, AstraZeneca Gothenburg Sweden

**Keywords:** gout, heart failure, hyperuricemia, urate‐lowering therapy

## Abstract

**Background:**

Elevated serum uric acid (sUA) is associated with heart failure (HF).

**Hypothesis:**

Urate‐lowering therapy (ULT) in HF is associated with lower risk of HF hospitalization (hHF) and mortality.

**Methods:**

Data on patients with HF and gout or hyperuricemia in the Clinical Practice Research Datalink database linked to the Hospital Episode Statistics and the Office for National Statistics in the United Kingdom were analyzed. Risks of hHF and all‐cause mortality or cardiovascular‐related mortality by ULT exposure (ULT initiated within ≤6 months of gout or hyperuricemia diagnosis) were analyzed in a propensity score‐matched cohort using adjusted Cox proportional hazards regression models.

**Results:**

Of 2174 propensity score‐matched pairs, patients were predominantly male, aged >70 years, with mean ± standard deviation sUA 9.3 ± 1.8 (ULT‐exposed) and 9.4 ± 1.9 mg/dL (ULT‐unexposed). At 5 years, ULT‐exposed patients had a 43% lower risk of hHF or all‐cause mortality (adjusted hazard ratio [HR]: 0.57; 95% confidence interval [CI]: 0.51–0.65) and a 19% lower risk of hHF or cardiovascular‐related mortality (adjusted HR: 0.81; 95% CI: 0.71–0.92) versus no ULT exposure.

**Conclusion:**

ULT was associated with reduced risk of adverse clinical outcomes in patients with HF and gout or hyperuricemia over 5 years.

## Practitioner points

- Raised serum uric acid is strongly associated with heart failure (HF); however, it is unknown whether urate‐lowering therapy (ULT) could help patients with HF.
- We assessed the relationship between ULT and HF outcomes using UK patient data, and found that the risk of the composite of hospital admission for HF or death was lower with ULT than with no ULT.
- These results support further investigation into the potential benefit of ULT in HF.

**Abbreviations**
CHD: chronic heart disease
CI: confidence interval
CPRD: Clinical Practice Research Datalink
CV: cardiovascular
eGFR: estimated glomerular filtration rate
HES: Hospital Episode Statistics
HF: heart failure
hHF: HF hospitalization
HR: hazard ratio
ITT: intention‐to‐treat
MACE: major adverse cardiac events
ONS: Office for National Statistics
RCT: randomized controlled trial
*SD*
: standard deviation
sUA: serum uric acid
ULT: urate‐lowering therapy
XOI: oxidase inhibitor

## 1 INTRODUCTION

Chronic heart failure (HF) is a major cause of poor quality of life and hospitalization, and has been associated with a 2‐year loss of life expectancy compared with the general population.^1^ HF is characterized by dyspnea, fatigue, and pulmonary congestion and/or peripheral edema due to fluid retention.^2^, ^3^

Hyperuricemia (serum uric acid [sUA] ≥6 mg/dL [females] and ≥7 mg/dL [males]) has been estimated to occur in more than half of patients (57%) with HF with preserved ejection fraction, and in 43% of patients with HF with reduced ejection fraction.^4^ Although elevated sUA has been strongly associated with endothelial and microvascular dysfunction and worse outcomes in HF,^5^, ^6^ it is unclear whether urate‐lowering therapy (ULT), currently indicated for gout, improves outcomes such as mortality in patients with HF.

The availability of real‐world data offers an opportunity to gain insights into the health outcomes associated with the use of ULT in patients with HF and gout or hyperuricemia, and the potential clinical benefit of extending the use of ULT within this patient group. In this study, our first objective was to characterize the patient population with HF and gout or hyperuricemia, who were exposed or unexposed to ULT, in terms of demographics, disease burden, comorbidities, and medication and healthcare use. Our second objective was to assess the relationship between ULT, HF hospitalization (hHF), and all‐cause or cardiovascular (CV)‐related mortality in patients with HF prescribed ULT for gout or hyperuricemia. We hypothesized that ULT would be associated with a lower risk of hHF and mortality.

## 2 METHODS

### 2.1 Study design and data source

This was an observational cohort study using secondary data from the UK‐based Clinical Practice Research Datalink (CPRD), an electronic health record database from primary care practices in the United Kingdom.^7^ CPRD data were linked^8^ to Hospital Episode Statistics (HES), a secondary care claims database in England with information on all outpatient and emergency care admissions to National Health Service hospitals in England, and to the Office for National Statistics (ONS) Death Registration Data including primary and contributing causes of death in England and Wales. No clinically identifiable patient information was made available to the researchers.

### 2.2 Study population

Patients aged ≥18 years at the index date with a previous hHF and a first‐ever recorded diagnosis of gout/elevated serum urate level (sUA >7 mg/dL for males and >6 mg/dL for females) during the enrollment period (January 1, 1997–June 30, 2019) were included.

Patients were excluded if they had no linked secondary care data, had gout or hyperuricemia diagnosed within 12 months before the initial diagnosis of gout within the enrollment period, had been prescribed ULT within 12 months before the first ever gout or hyperuricemia diagnosis, had a malignancy diagnosis within 12 months before the index date, or had at least two estimated glomerular filtration rate (eGFR) test measurements <30 mL/min/1.73 m^2^ during the baseline period.

Two partially overlapping cohorts were considered: (1) for objective 1, all eligible patients were stratified by ULT initiation within 6 months of incident gout or hyperuricemia, with their index date as the date of the initial gout or hyperuricemia diagnosis; and (2) for objective 2, propensity score matching in accrual blocks was used to identify ULT initiators and matched non‐initiators with corresponding index dates. For the ULT initiators, the date of ULT initiation was the index date, while a random date within the same 6‐month block was selected for the non‐initiators (see statistical analysis below). All patients were followed until the date of death, transfer out of practice, or the last day of available data, whichever occurred first.

### 2.3 Variables

Baseline characteristics ascertained during the 1 year before the index date are listed in **Supporting Information: Baseline characteristics**.

### 2.4 ULT exposure

Details of ULTs included in this analysis (allopurinol, febuxostat, probenecid, lesinurad, pegloticase, and lesinurad/allopurinol) were extracted from primary care prescription data. ULT was initiated (index date) on the date of the first ULT prescription issued by a general practitioner. Among ULT initiators, patterns of ULT use in the year following initiation were recorded and included the occurrence and timing of a switch to another ULT, defined as prescription of another ULT, as well as of ULT discontinuation (last prescription issued).

### 2.5 Clinical outcomes

Clinical outcome events (hHF, CV‐related mortality, and all‐cause mortality) were assessed during the 5 years following the index date. hHF was defined as hospitalization with a primary International Classification of Diseases Tenth Revision diagnosis code of I50.xx, extracted from HES data. Mortality data were extracted from the ONS mortality file. Conditions underlying CV‐related mortality included myocardial infarction, stroke or transient ischemic attack, HF, or unstable angina.

### 2.6 Statistical analysis

Baseline characteristics were described; continuous variables were expressed as mean ± standard deviation (*SD*), and binary or categorical variables were expressed as *n* (%).

An “on‐treatment” approach was used as the main comparative analysis due to extensive ULT switching and discontinuation (Figure S1), in which patients were censored on the first treatment switch. The intention‐to‐treat (ITT) population was used for the sensitivity analysis, in which patient outcomes following treatment switch were included.

**Figure 1:**
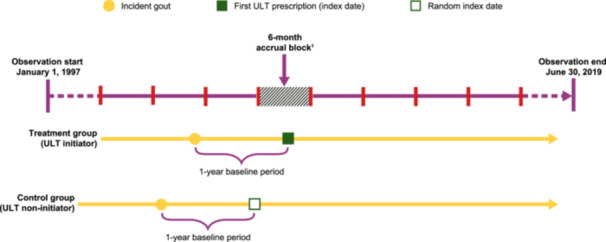
Creation of accrual blocks and assignment of index dates. ^1^Initiators and non‐initiators were identified within the same accrual block. ULT, urate‐lowering therapy.

A stepwise approach was used to confirm that the two comparator groups could be balanced by potential confounders and that the available data would provide sufficient power for the planned comparisons of the risk of hHF and mortality events between ULT initiators and non‐initiators (see **Supporting Information: Propensity score matching** and Figure 1). The balancing of baseline covariates through propensity score matching has been described in full previously.^9^

A propensity score‐matched dataset was established, then cumulative incidence curves for composite hHF or all‐cause mortality and composite hHF or CV‐related mortality were calculated using a time‐to‐first‐event analysis both for ULT initiators and ULT non‐initiators. Associations between treatment with ULT and the composite events were evaluated using adjusted Cox proportional hazard regression models and expressed as a hazard ratio (HR) with 95% confidence interval (CI). Multiple imputation was used for missing data. All statistical analyzes were performed using R software (version 4.0.4; R Foundation for Statistical Computing, Vienna, Austria).

### 2.7 Ethics

The CPRD has ethics approval from the Health Research Authority to support research using anonymized patient data (IRAS ID: 242149).

## 3 RESULTS

### 3.1 ULT initiation within 6 months of incident gout or hyperuricemia

**Figure 2:**
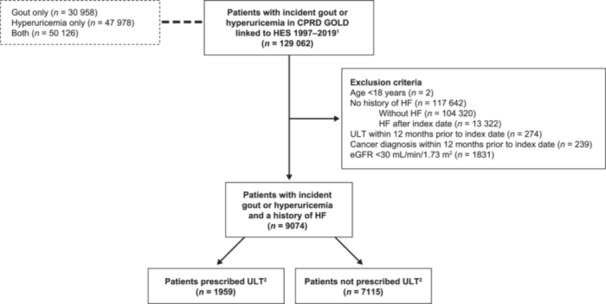
Patients meeting study inclusion criteria. ^1^Patients were required to have been registered with a general practitioner for at least 1 year. ^2^Patient exposure with ULT was defined as ULT prescription within the first 6 months after incident gout or hyperuricemia. CPRD, Clinical Practice Research Datalink; eGFR, estimated glomerular filtration rate; HES, Hospital Episode Statistics; HF, heart failure; ULT, urate‐lowering therapy.

A total of 9074 patients with HF and incident gout or hyperuricemia were identified during the study period. To study the first objective, we compared the 1959 (21.6%) patients who were prescribed ULT within the first 6 months after incident gout or hyperuricemia with those who were not (7115 [78.4%] patients; Figure 2).

Baseline patient characteristics in the unmatched cohort are shown in **Supporting Information: Baseline characteristics (unmatched cohort)** and Table S1. The patterns of ULT use following initiation within 6 months are shown in Table 1. The mean ± *SD* time from diagnosis to the first ULT prescription was 43.4 ± 45.9 days. In the first year after ULT initiation, patients received eight prescriptions, and the mean duration of ULT treatment was 272 days, corresponding to 70% of prescribed days in the first year following initiation. Allopurinol was the most common ULT initiated. A total of 403/709 (57%) ULT‐exposed patients and 519/2038 (25%) ULT‐unexposed patients achieved the sUA target (≤7 mg/dL [males] and ≤6 mg/dL [females]) within 5 years of ULT initiation.

**Table 1:** ULT patterns in the year following initiation in patients exposed to ULT within 6 months of the index date (unmatched cohort).

Patterns of ULT	ULT‐exposed patients		
	*N* = 1959		
	*n* (%) or mean ± *SD*	Range	Median (IQR)
Number of patients taking ULT type at least once			
Allopurinol	1949 (99.5)	–	–
Probenecid	6 (0.3)	–	–
Sulfinpyrazone	4 (0.2)	–	–
Febuxostat	21 (1.1)	–	–
Number of prescriptions by type (in those taking that type)			
Allopurinol	8.4 ± 7.2	1–67	7 (3–13)
Probenecid	2.2 ± 1.3	1–4	2 (1–3)
Sulfinpyrazone	4.0 ± 3.2	1–8	3.5 (1.8–5.8)
Febuxostat	6.8 ± 11.3	1–53	4 (1–6)
Duration of ULT^a^ (days)	272 ± 127	1–365	365 (152–365)
Average ULT daily dose (mg)			
Allopurinol	188 ± 85	50–573	175 (100–289)
Probenecid	979 ± 614	500–2000	750 (500–1281)
Sulfinpyrazone	232 ± 128	150–420	178 (155–255)
Febuxostat	81 ± 6	80–107	80 (80–80)
Number of ULT prescriptions	8 ± 7	1–67	7 (4–13)
Days supplied^b^	284 ± 160	1–1008	336 (140–392)
Proportion of days covered (from ULT initiation to 1 year after gout/hyperuricemia)	0.7 ± 0.3	0–1	0.9 (0.4–1)
Number of patients who achieved sUA targets	466 (24.0)	–	–
Time from incident gout/hyperuricemia to ULT initiation (days)	43.4 ± 45.9	0–183	25.0 (8.0–64.0)

Abbreviations: HF, heart failure; IQR, interquartile range; *SD*, standard deviation; ULT, urate‐lowering therapy.

^a^ Number of days from the date of ULT initiation to the date of the last prescription plus the number of days' supply. This measure includes periods of time when ULT was not administered and allows for gaps in therapy in the calculation of duration.

^b^ Number of days that ULT was prescribed.

### 3.2 Baseline patient characteristics and treatment of the propensity score‐matched cohort

For the second objective, the requirement for ULT treatment within 6 months of incident gout was dropped, allowing a total of 2174 propensity score‐matched pairs with and without ULT prescription at the index date to be identified for analysis. Baseline characteristics in the propensity score‐matched cohort were mostly balanced across ULT‐exposed and ULT‐unexposed patients, with a standardized difference of >10% (Table S2). Overall, patients were predominantly male and aged >70 years. For the ULT‐exposed versus ULT‐unexposed groups, mean ± *SD* body mass index was 29.7 ± 6.1 and 29.2 ± 6.1 kg/m^2^ and mean ± *SD* sUA was 9.3 ± 1.8 and 9.4 ± 1.9 mg/dL, respectively. Overall, patients in the propensity score‐matched cohort had no hHF within 1 year before index; the most common comorbidities were hypertension (>90% of patients), chronic kidney disease (>50% of patients), and diabetes (>20% of patients).

### 3.3 ULT use and risk of negative health outcomes

**Figure 3:**
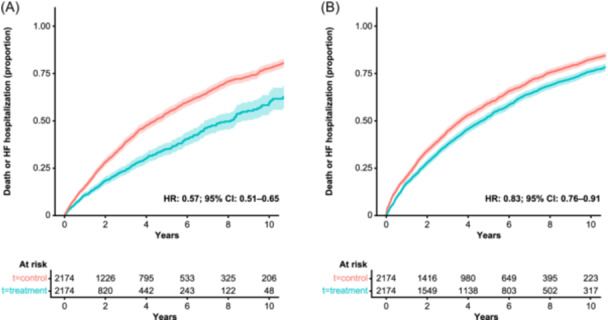
Cumulative incidence curves for the composite endpoint HF hospitalization or all‐cause death, stratified by ULT exposure, in the (A) on‐treatment and (B) intention‐to‐treat patient population. Propensity score models were adjusted for categorical variables (flare in previous 12 months; male sex; geographical region; urban location; index of multiple deprivation quintiles; currently smokes; currently drinks alcohol; presence of tophi, hypertension, diabetes, chronic kidney disease, myocardial infarction, hyperlipidemia; use of statin, fibrate, angiotensin‐converting enzyme agonist, mineralocorticoid, neprilysin/renin‐angiotensin‐aldosterone system inhibitors combination, beta‐blockers, calcium channel blockers, colchicine, aspirin, non‐steroidal anti‐inflammatories, or diuretics) and for continuous variables (age; body mass index; estimated glomerular filtration rate; serum uric acid; albumin; cholesterol; years since HF; number of HF hospitalizations in the past 5 years; Charlson Comorbidity Index; number of primary care visits; length of stay for hospitalizations in the previous year; days spent in hospital in the previous year; years from incident gout diagnosis or hyperuricemia to index date; number of serum uric acid measurements in the previous year; and creatinine). Shaded bands indicate pointwise 95% CIs. CI, confidence interval; HF, heart failure; HR, hazard ratio; ULT, urate‐lowering therapy.

Cumulative incidence of the composite of hHF or all‐cause mortality over 5 years of follow‐up was lower in patients who were exposed to ULT compared with those who were not (Figure 3A). Following adjusted analysis, ULT treatment was associated with a 43% lower risk of the composite endpoint (HR: 0.57; 95% CI: 0.51–0.65; Figure 3A). Cumulative incidence curves for the components of the composite endpoint indicated there were lower event rates for both all‐cause mortality and hHF in patients receiving ULT, with the largest difference seen with all‐cause mortality (Figure S2A). The ITT sensitivity analysis indicated that ULT was associated with a 17% lower risk of the composite of hHF or all‐cause mortality (HR: 0.83; 95% CI: 0.76–0.91; Figure 3B) and that this was also driven by the lower risk of all‐cause mortality with ULT exposure (Figure S2B).

**Figure 4:**
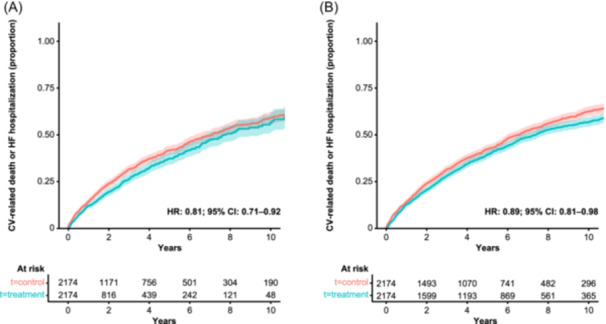
Cumulative incidence curves for the composite endpoint HF hospitalization or CV‐related death, stratified by ULT exposure, in the (A) on‐treatment and (B) intention‐to‐treat patient population. Propensity score models were adjusted for categorical variables (flare in previous 12 months; male sex; geographical region; urban location; index of multiple deprivation quintiles; current smokes; current drinks alcohol; presence of tophi, hypertension, diabetes, chronic kidney disease, myocardial infarction, hyperlipidemia; use of statin, fibrate, angiotensin‐converting enzyme agonist, mineralocorticoid, neprilysin/renin‐angiotensin‐aldosterone system inhibitors combination, beta‐blockers, calcium channel blockers, colchicine, aspirin, non‐steroidal anti‐inflammatories, or diuretics) and for continuous variables (age; body mass index; estimated glomerular filtration rate; serum uric acid; albumin; cholesterol; years since HF; number of HF hospitalizations in the past 5 years; Charlson Comorbidity Index; number of primary care visits; length of stay for hospitalizations in the previous year; days spent in hospital in the previous year; years from incident gout diagnosis or hyperuricemia to index date; number of serum uric acid measurements in the previous year; and creatinine). Shaded bands indicate pointwise 95% CIs. CI, confidence interval; CV, cardiovascular; HF, heart failure; HR, hazard ratio; ULT, urate‐lowering therapy.

Cumulative incidence of the composite of hHF or CV‐related mortality events over 5 years of follow‐up was lower in patients exposed to ULT than in those who were not (Figure 4A). Following adjusted analysis, ULT was associated with a 19% lower risk of the composite endpoint hHF or CV‐related mortality (HR: 0.81; 95% CI: 0.71–0.92; Figure 4A). In the ITT sensitivity analysis, ULT was associated with an 11% lower risk of hHF or CV‐related mortality (HR: 0.89; 95% CI: 0.81–0.98; Figure 4B).

## 4 DISCUSSION

The aim of this analysis of real‐world data from primary and secondary care was to assess the impact of ULT on the risk of adverse health outcomes in patients with HF and gout or hyperuricemia. We found that ULT was associated with a reduced risk of hHF and all‐cause mortality, as well as of hHF and CV‐related mortality, over 5 years of follow‐up in patients with HF and gout or hyperuricemia. An on‐treatment approach was applied in the analyzes to account for extensive treatment switching, and a propensity score‐matched cohort of initiators and non‐initiators of ULT was assessed in 6‐month accrual blocks to account for potential confounding variables and clinical practice changes over the study period.

In this analysis, allopurinol was the most commonly prescribed agent, prescribed at a dosage consistent with the clinical pattern of ULT in patients with gout or hyperuricemia reported elsewhere.^10^, ^11^, ^12^ The mean daily dose of 188 mg observed in our analysis was within the range recommended for mild gout^13^ but lower than that typically studied in clinical trials.^14^, ^15^

The duration of ULT was 70% of the first year following initiation. The target sUA (≤7 mg/dL for males and ≤6 mg/dL for females) was achieved in more than half (57%) of ULT‐exposed patients within 5 years, a higher proportion than previously reported in another real‐world study (range: 32%–51%), in which, notably, the target of ≤6 mg/dL was used for both males and females.^10^, ^16^

In this analysis of data on clinical practice, ULT was associated with a lowered risk of hHF and all‐cause mortality, as well as of hHF and CV‐related mortality in patients with HF and gout or hyperuricemia. Our findings contrast with previous observational data from a study investigating a small group of patients with HF with preserved ejection fraction and hyperuricemia (*n* = 254), in which there were no significant differences in cardiac and all‐cause mortalities between those who received ULT and those who did not.^6^ Although raised sUA has been identified as an independent predictor of poorer outcomes in HF,^5^, ^6^ data from clinical trials remain unclear on the benefit of ULT on HF‐related outcomes in patients with hyperuricemia. One meta‐analysis found that allopurinol and febuxostat were not associated with a significant reduction in major adverse cardiac events (MACE) compared with placebo or no ULT,^17^ whereas in another study ULT did not reduce the risk of either MACE or all‐cause mortality.^18^ Results were similarly inconclusive in a meta‐analysis of patients with hyperuricemia and different CV risk. In those with low CV risk (i.e., patients with no previous HF, atrial fibrillation, chronic heart disease [CHD], myocardial infarction, cerebrovascular accident, or transient ischemic attack), five studies showed that, compared with no treatment or placebo, xanthine oxidase inhibitors (XOIs) were associated with a lower risk of both new‐onset CV events (e.g., arrhythmia, HF, CHD, peripheral vascular disease) and MACE. By contrast, two studies indicated that XOIs lowered the risk of new‐onset CV events but not MACE in patients with high CV risk.^19^

Variability in study designs and analytical approaches may have contributed to inconsistencies in study findings and uncertainty over the CV benefit of ULT.^20^ Differences in drug mechanism of action and patient phenotype may also need to be considered when interpreting real‐world data on ULT and the risk of CV outcomes.^21^ Although there are plausible biological mechanisms by which ULT could reduce the risk of CV‐related outcomes, including lowering systemic inflammation, oxidative stress, and hyperuricemia‐associated endothelial dysfunction,^22^ there remains a need for large, well‐designed, placebo‐controlled, randomized controlled trials (RCTs) to demonstrate the CV effect of ULT and generate results applicable to clinical practice.

Our analysis has several strengths. The CPRD dataset comprises ~4.4 million active patients (6.9% of the UK population) and the patients included in the analysis were broadly representative of the general population in terms of age, sex, and ethnicity. To account for changes in clinical practice during the long enrollment period, 6‐month accrual blocks were applied, during which ULT initiators and non‐initiators were identified. A predefined stepwise analysis plan was used. The exposure was assessed in the first step, with the degree of switching also examined to understand the implication of an ITT versus an on‐treatment analysis approach in this population. Because treatment switching was common, the on‐treatment approach was considered more robust, and ITT was used for the sensitivity analysis. This strengthened our results and ensured sufficient statistical power. Thereafter, assessment of the primary outcome in the whole population and exposure time confirmed the comparative analysis was statistically sound based on statistical power and the groups could be balanced with respect to morbidity and background characteristics with propensity score matching.

Study limitations included the potential for inaccurate recording of information and for missing data on certain confounders that are inherent in database studies. Most patients received allopurinol and it was not possible to assess the impact of individual ULTs. Our findings may be generalizable only to patients receiving allopurinol ≤300 mg, and higher doses could have a different impact on the study outcomes. Primary care data were used to ascertain whether a patient received a ULT prescription; the unavailability of data on ULT received through secondary care could mean that some patients were misclassified as ULT‐unexposed. Although the use of loop diuretics was controlled at baseline, changes in diuretic medication over the course of study follow‐up could have been a significant confounder; loop or thiazide diuretics have previously been reported to increase the risk of incident gout.^23^ Our study was not powered to detect differences in HF‐specific outcomes, and we only assessed differences in composite outcomes of hHF and mortality.

## 5 CONCLUSION

Using longitudinal primary care data representative of the UK patient population, ULT was found to be associated with a lower risk of hHF or all‐cause mortality, as well as of hHF or CV‐related mortality, in patients with HF and gout or hyperuricemia. These results are consistent with the hypothesis that lowering sUA may improve outcomes in patients with HF and/or gout or hyperuricemia, and support further investigation into the potential benefit of ULT in HF in prospective RCTs.

## AUTHOR CONTRIBUTIONS

Steven J. Kiddle, Karolina Andersson Sundell, Shira Perl, Stephen Nolan, and Magnus Bjursell contributed to the conception and design of the study as well as to the acquisition, analysis, and interpretation of the data. All authors drafted or critically revised this article for important intellectual content and approved the final version. Steven J. Kiddle agrees to be accountable for all aspects of the work.

## CONFLICT OF INTEREST STATEMENT

All authors were employees and stockholders of AstraZeneca at the time of the analysis and manuscript development.

## Supporting information

Supporting information.

## Data Availability

Data underlying the findings described in this manuscript are accessible to approved researchers, who are covered by a CPRD data license, for approved purposes.
