# Supplementary material for: Urate‐lowering therapy in patients with hyperuricemia and heart failure: A retrospective cohort study using the UK Clinical Practice Research Datalink
Source: Clin Cardiol. 2024 Jun 14;47(6):e24297. doi: 10.1002/clc.24297 (PMC11177015; doi:10.1002/clc.24297)
Supplement: Supplementary file 1 — Supporting information. [file CLC-47-e24297-s001.docx]

Urate-lowering therapy in patients with hyperuricemia and heart failure: A retrospective cohort study using the UK Clinical Practice Research Datalink

Steven J. Kiddle, PhD^1^, Karolina Andersson Sundell, PhD^2^, Shira Perl, MD^3^, Stephen Nolan, PhD^4^, Magnus Bjursell, PhD^5^

^1^Data Science & Advanced Analytics, Data Science & Artificial Intelligence, R&D, AstraZeneca, Cambridge, UK; ^2^Cardiovascular, Renal and Metabolic (CVRM) Evidence, BioPharmaceuticals Medical, AstraZeneca, Gothenburg, Sweden; ^3^Late-stage Development, Clinical, Cardiovascular, Renal and Metabolic (CVRM), BioPharmaceuticals R&D, AstraZeneca, Gaithersburg, Maryland, USA; ^4^Late-stage Development, Clinical, Cardiovascular, Renal and Metabolic (CVRM), BioPharmaceuticals R&D, AstraZeneca, Cambridge, UK; ^5^Global Medical Affairs, Clinical, Cardiovascular, Renal and Metabolic (CVRM), BioPharmaceuticals Medical, AstraZeneca, Gothenburg, Sweden

**Correspondence:** Steven J. Kiddle, Data Science & Advanced Analytics, Data Science & Artificial Intelligence, R&D, AstraZeneca, Cambridge, UK.
Tel: +44 (0) 1223 35841
Email: [steven.kiddle@astrazeneca.com](mailto:steven.kiddle@astrazeneca.com)

Contents

[Supporting information: Baseline characteristics 3](#_Toc167455945)

[Supporting information: Propensity score matching 4](#_Toc167455946)

[Supporting Information: Baseline characteristics (unmatched cohort) 6](#_Toc167455947)

[Supporting Information: Reference 7](#_Toc167455948)

[Table S1. Baseline patient characteristics at index stratified by ULT status (unmatched cohort). 8](#_Toc167455949)

[Table S2. Baseline patient characteristics by ULT exposure at index date following propensity score matching. 10](#_Toc167455950)

[Figure S1. Cumulative incidence curves for treatment switching after the index date^1^ (propensity score-matched cohort) 13](#_Toc167455951)

[Figure S2. Cumulative incidence curves for the components of the composite endpoint of HF hospitalization or all-cause death in the (A) on‑treatment and (B) intention-to-treat patient populations 14](#_Toc167455952)

# Supporting information: Baseline characteristics

Key data extracted on baseline characteristics included the following: patient demographics; HF classification (International Classification of Diseases Tenth Revision diagnosis code I50.xx, I11.0, I13.0, I13.2, I26.0; or Ninth Revision diagnosis code 428.0, 428.1, 428.9) on the most recent visit for HF prior to the index date; the number of HF hospitalizations during the 1 year prior to the index date; gout diagnosis, gout medication, and sUA at the index date; comorbidities during the 5 years prior to baseline; medications based on prescriptions during the 1 year prior to the index date; and medical resource use during the 1 year prior to the index date.

# Supporting information: Propensity score matching

First, patient accrual blocks and index dates were established. The time between January 1, 1997, and June 30, 2019, was split into 6-month accrual blocks, within which ULT initiators were assigned to the treatment group and ULT non-initiators were assigned to the control group. The index date was the date of the first prescription for ULT for patients in the treatment group. In the control group, the index date was randomly selected from all time points within the 6-month accrual block, at which point the patient was eligible to enter the study (**Figure 1**).

Second, potential confounders were identified and assessed for imbalance between the two groups by determining the number and percentage of omissions for each variable at baseline overall and per treatment group. Confounders highly associated with each other were either combined or were represented by just one.

Third, propensity score matching was applied. Propensity scores (the predicted probability of being assigned to a treatment) were estimated separately for each accrual block using logistic regression with ULT initiation as the outcome. For continuous variables, four knot splines were used or the highest knot number that could be fitted within the relevant accrual block (based on degrees of freedom). All relevant baseline confounders were incorporated as covariates in the logistic regression. Propensity score methods and adjustment for a broad range of potential confounders were used to minimize indication bias, where patients prescribed ULT were likely to have more severe gout or higher sUA and to be at increased risk of adverse events than those who were not. Following estimation of the propensity scores, ULT-treated and ULT-untreated patients were matched within each accrual block using the logit of the propensity score and a ‘greedy’ nearest-neighbor approach.

Fourth, the degree of overlap between the treated and untreated groups in the logit of the propensity score was assessed by comparing the standardized differences before and after matching to allow redefinition of propensity score models where the overlap was poor. Once sufficiently balanced groups were established, the total number of events was calculated to estimate power. A total of 1189 matched pairs (1:1 matched) were calculated to be required to detect a hazard ratio of 0.85 with at least 80% power using a two-sided significance level of 0.05, assuming a 5-year risk of outcome event of 50%.^1^

Fifth, the degree of treatment switching was assessed descriptively using Kaplan–Meier curves to plot the time to switching to a different treatment in the treatment group.

# Supporting Information: Baseline characteristics (unmatched cohort)

Overall, patients were predominantly male and aged >70 years. For the ULT‑exposed versus ULT-unexposed groups, mean ± standard deviation body mass index was 30.0 ± 6.3 and 28.7 ± 5.9 kg/m^2^, mean ± *SD* sUA was higher (9.6 ± 1.8 vs. 8.3 ± 1.7 mg/dL), and a higher proportion of patients had a gout diagnosis at index (52.3% vs. 43.5%), respectively. For the unmatched cohort, there were 4.3 ± 5.0 and 4.6 ± 9.5 hHFs within 5 years prior to index in the ULT-exposed and ULT-unexposed groups, respectively. Overall, the most common comorbidities over the 5-year baseline period were hypertension (>90% of patients), diabetes (>20% of patients), and hyperlipidemia (>17% of patients).

# Supporting Information: Reference

1. Taylor CJ, Ordonez-Mena JM, Roalfe AK, et al. Trends in survival after a diagnosis of heart failure in the United Kingdom 2000-2017: population based cohort study. *BMJ*. 2019;364:l223.

Table S1. Baseline patient characteristics at index stratified by ULT status (unmatched cohort).

| **Variable** | **ULT-exposed (*N* = 1959)** | **ULT-unexposed (*N* = 7116)** |
| --- | --- | --- |
| Male | 1277 (65.2) | 4197 (59.0) |
| Age at index date (years) | 74.0 ± 10.8 | 76.5 ± 10.4 |
| From most deprived quintile | 542 (27.7) | 1559 (21.9) |
| BMI (kg/m^2^) | 30.0 (6.3) | 28.7 (5.9) |
| Prior HF-related factors |  |  |
| Years since index HF | 5.0 ± 4.6 | 5.0 ± 4.6 |
| Number of HF hospitalizations^a^ | 4.3 ± 5.0 | 4.6 ± 9.5 |
| Resource utilizations during 1 year before the index date | | |
| Hospital admission for HF | 881 (45.0) | 3210 (45.1) |
| Number of HF hospitalizations | 1.1 ± 1.9 | 1.2 ± 2.8 |
| Use of mechanical ventilation | 2 (0.1) | 15 (0.2) |
| Gout-related factors |  |  |
| Gout diagnosis at index date | 1024 (52.3) | 3097 (43.5) |
| Baseline serum urate level at index date, mg/dL | 9.6 ± 1.8 | 8.3 ± 1.7 |
| Laboratory measurements (1-year baseline period) | | |
| Glomerular filtration rate (mL/min/1.73 m^2^) | 51.4 ± 18.5 | 52.7 ± 16.4 |
| Cholesterol (mg/dL) | 179.0 ± 46.0 | 177.1 ± 45.1 |
| Creatinine (mg/dL) | 1.3. ± 0.3 | 1.3. ± 0.3 |
| Comorbidities (5-year baseline period) |  |  |
| CCI (excluding HF) | 2.1 ± 1.5 | 2.2 ± 2.0 |
| Diabetes | 403 (20.6) | 1428 (20.1) |
| Hypertension | 1859 (94.9) | 6612 (92.9) |
| Prior MI | 279 (14.2) | 930 (13.1) |
| Hyperlipidemia (using ICD codes) | 349 (17.8) | 1254 (17.6) |
| Medication use (1-year baseline period) |  |  |
| Calcium channel blockers | 448 (22.9) | 1741 (24.5) |
| Colchicine | 395 (20.2) | 1090 (15.3) |
| Diuretics | 1851 (94.5) | 6378 (89.6) |
| Mineralocorticoid | 599 (30.6) | 1648 (23.2) |
| NSAIDs | 927 (47.3) | 2922 (41.1) |
| All-cause medical resource use during 1-year baseline period | | |
| Number of primary care visits (per patient) | 15.3 ± 11.7 | 14.7 ± 11.8 |
| Had primary care visits | 1941 (99.1) | 7057 (99.2) |

Abbreviations: BMI, body mass index; CCI, Charlson Comorbidity Index; HF, heart failure; ICD, International Classification of Diseases; MI, myocardial infarction; NSAID, non-steroidal anti-inflammatory drug; *SD*, standard deviation; ULT, urate-lowering therapy

Continuous variables are presented as means ± *SD*; binary and categorical variables are *n* (%).

^a^5 years before the index date.

Table S2. Baseline patient characteristics by ULT exposure at index date following propensity score matching.

| Variable | ULT-exposed *N* = 2174 | ULT-unexposed *N* = 2174 | Standardized mean difference |
| --- | --- | --- | --- |
| Male | 1420 (65.3) | 1405 (64.6) | 0.01 |
| Age at index date, years | 75 ± 10.3 | 76 ± 10.3 | 0.10 |
| Body mass index (kg/m^2^) | 29.7 ± 6.1 | 29.2 ± 6.1 | 0.07 |
| Underweight (15–18.5) | 14 (0.6) | 21 (1) | 0.10 |
| Healthy weight (18.5–25) | 399 (18.4) | 442 (20.3) | - |
| Overweight (25–30) | 697 (32.1) | 656 (30.2) | - |
| Obese (30+) | 784 (36.1) | 696 (32) | - |
| Missing | 280 (12.9) | 359 (16.5) | - |
| **Prior HF-related factors** |  |  |  |
| Hospital admission for HF (1 year before index date) | 150 (6.9) | 163 (7.5) | 0.09 |
| Number of HF hospitalizations (1 year before index date) | 0.1 ± 0.5 | 0.1 ± 0.5 | 0.04 |
| Use of mechanical ventilation | 5 (0.2) | 2 (0.1) | 0.03 |
| **Gout-related factors** |  |  |  |
| Gout flare in the previous 12 months | 1279 (58.8) | 1241 (57.1) | 0.04 |
| Baseline serum urate level at index date (mg/dL) | 9.3 ± 1.8 | 9.4 ± 1.9 | 0.05 |
| **Laboratory measurements (most recent before index date)** |  |  |  |
| Glomerular filtration rate (mL/min/1.73 m^2^) | 51.0 ± 18.0 | 51.0 ± 16.2 | <0.01 |
| Cholesterol (mg/dL) | 176.3 ± 45.3 | 175.4 ± 45.4 | 0.02 |
| Creatinine (mg/dL) | 1.3 ± 0.3 | 1.3 ± 0.3 | 0.06 |
| **Comorbidities (within 5 years before the index date)** |  |  |  |
| Diabetes | 481 (22.1) | 490 (22.5) | 0.01 |
| Hypertension | 2085 (95.9) | 2062 (94.8) | 0.05 |
| Chronic kidney disease | 1189 (54.7) | 1153 (53) | 0.03 |
| Prior MI | 269 (12.4) | 275 (12.6) | <0.01 |
| Hyperlipidemia (using ICD codes) | 376 (17.3) | 406 (18.7) | 0.04 |
| CCI (excluding heart failure) | 2.3 ± 2.0 | 2.3 ± 2.0 | <0.01 |
| **Medication use (during the 1-year baseline period)** |  |  |  |
| Statins | 1206 (55.5) | 1162 (53.4) | 0.04 |
| Fibrates | 18 (0.8) | 29 (1.3) | 0.05 |
| Calcium channel blockers | 497 (22.9) | 456 (21) | 0.05 |
| Aspirin | 1069 (49.2) | 1076 (49.5) | <0.01 |
| Insulin | 143 (6.6) | 151 (6.9) | 0.02 |
| NSAIDs | 1142 (52.5) | 1093 (50.3) | 0.05 |
| Colchicine | 845 (38.9) | 801 (36.8) | 0.04 |
| Corticosteroids | 452 (20.8) | 378 (17.4) | 0.09 |
| Diuretics | 2035 (93.6) | 2004 (92.2) | 0.06 |
| Beta-blockers | 1082 (49.8) | 1045 (48.1) | 0.03 |
| Angiotensin-converting enzyme agonist | 1799 (82.8) | 1783 (82) | 0.02 |
| Mineralocorticoid | 637 (29.3) | 616 (28.3) | 0.02 |
| Neprilysin/RAASi combination | 1 (0) | 0 (0) | - |
| **All-cause medical resources use (during the1-year baseline period)** |  |  |  |
| Number of primary care visits (per patient) | 16.4 ± 12.4 | 15.8 ± 13.1 | 0.05 |
| Had primary care visits | 2155 (99.1) | 2158 (99.3) | 0.02 |
| Number of hospital in-patient visits | 0.9 ± 1.5 | 1 ± 2.5 | 0.05 |
| Inpatient length of stay, total days | 11.6 ± 18.7 | 12.9 ± 21.2 | 0.07 |
| Hospitalization | 982 (45.2) | 996 (45.8) | 0.01 |

*Note:* Continuous variables are presented as mean ± *SD*; binary and categorical variables are *n* (%). Index date was defined as the date of initiation of ULT for ULT-exposed patients and the date of the initial gout diagnosis for unexposed patients.

Abbreviations: BMI, body mass index; CCI, Charlson Comorbidity Index; HF, heart failure; ICD, International Classification of Diseases; MI, myocardial infarction; *n*, number; NSAID, non-steroidal anti-inflammatory drug; RAASi, renin-angiotensin-aldosterone system inhibitor; *SD*, standard deviation; ULT, urate-lowering therapy.

Figure S1. Cumulative incidence curves for treatment switching after the index date^1^ (propensity score-matched cohort)

2111

2111

ULT, urate-lowering therapy.

^1^Data censored by death, patient transferring out of practice, practice no longer sending data, or end of the follow-up period. Treatment switch was defined as first or last ULT prescription date during follow-up for control and treatment patients, respectively.

Figure S2. Cumulative incidence curves for the components of the composite endpoint of HF hospitalization or all-cause death in the (A) on‑treatment and (B) intention-to-treat patient populations


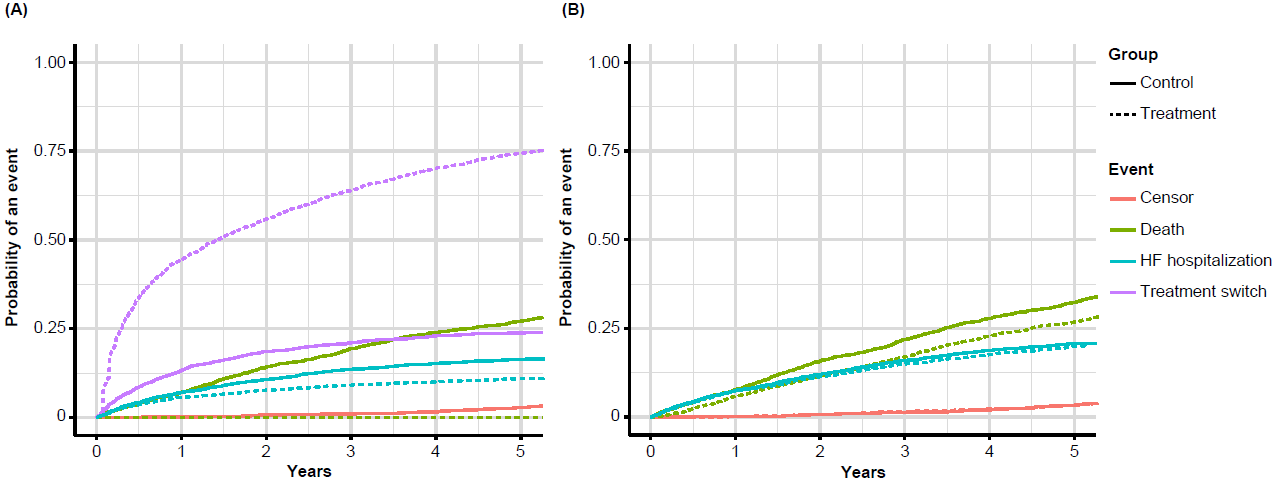


HF, heart failure.
